# Effects of clear aligners treatment in growing patients: a systematic review

**DOI:** 10.3389/froh.2024.1512838

**Published:** 2025-01-06

**Authors:** Vincenzo D’Antò, Vittoria De Simone, Silvia Caruso, Paolo Bucci, Rosa Valletta, Roberto Rongo, Rosaria Bucci

**Affiliations:** ^1^ Department of Neurosciences, Reproductive Sciences and Oral Sciences, Section of Orthodontics and Temporomandibular Disorders, University of Naples Federico II, Naples, Italy; ^2^ Department of Life, Health and Environmental Sciences, University of L'Aquila, L'Aquila, Italy; ^3^ Department of Public Health, Section of Hygiene, University of Naples Federico II, Naples, Italy

**Keywords:** clear aligners appliance, mixed dentition, early treatment, interceptive treatment, functional orthodontic

## Abstract

**Introduction:**

In recent years, the use of Clear aligners (CA) has been diffused among children and adolescents. This systematic review aimed to summarize the literature regarding the effects of CA therapy in growing patients, including dentoalveolar and skeletal effects, periodontal changes, and quality of life measurements.

**Methods:**

An electronic search on four databases was performed until September 2023, and studies including patients <18 years, treated with CA were selected. Studies with less than 10 patients and *in vitro*/laboratory studies were excluded. Study selection, data extraction, and risk of bias assessment were independently performed by two reviewers. The “Risk of Bias 2” (RoB 2) and the “Risk of Bias in Non-randomized Studies of Interventions” (ROBINS-I) tools were applied to assess the methodological quality of the included studies. Due to the heterogeneity in methodologies and outcomes encountered in the included studies, a qualitative synthesis of the results was provided.

**Results and conclusions:**

The search resulted in 32 papers (3 RCTs), with sample sizes ranging between 15 and 113. The overall risk of bias in the RCT was low, while the risk of bias in the non-RCT ranged between moderate and serious for most of the included studies. Dentoskeletal and periodontal effects were the most frequently reported outcomes. The most common comparison group was multibracket fixed therapy, while only 5 studies had an untreated control group. Significant changes in the transversal maxillary arch width after treatment with CA were reported in some studies. However, while dentoalveolar effects have been reported consistently, controversial findings were found regarding the changes in skeletal bases after treatment with CA. Concerning the results on the sagittal plane, the current literature does not support the effectiveness of CA with mandibular advancement features in correcting dentoskeletal Class II, compared to traditional functional orthopedic appliances. In the short-term evaluation, periodontal variables and bacterial levels seemed to be better controlled during CA therapy, compared to the fixed multibracket therapy. With regard to quality of life measurements, there are inconsistent findings to support differences between CA therapy and fixed multibracket appliances. Nevertheless, additional high-quality studies are required to formulate more reliable conclusions.

**Systematic Review Registration:**

https://osf.io/wmerq.

## 1 Introduction

Several orthodontic problems should already be treated at an early age to prevent the necessity of future complex and expensive procedures (1). The primary goals of early orthodontic interventions are to prevent or reduce the developmental of dentoskeletal abnormalities, to maintain space following premature loss of deciduous teeth, to manage functional habits that could contribute to malocclusion, and to minimize the invasiveness of the second treatment phase (2).

Previous studies have demonstrated that appliance acceptability, social impact, and quality of life, represent key elements in achieving good patient compliance and to improve treatment efficacy (3–5). Furthermore, it has been recognized that patient's aspect with orthodontic appliance could affect physical, social, and psychological well-being (6).

In this context, orthodontic devices with limited aesthetic impact, such as clear aligners (CA), observed a huge increase in their use in the last decades (7–9). As a result, there has been a substantial expansion in research focused on CA treatment (10). Possible advantages of these devices include the ability to remove the appliance during meals and oral hygiene procedures, and the reduction in the pain levels experienced by patients (11).

For many years, orthodontic treatments with CA were directed only to adult patients with full permanent dentition, with the aim to treat mild to moderate malocclusions (12). Over time, with the evolution of technologies and the improvement of material properties, the indications for CA use have increased, and this treatment approach has been extended also to more complex cases (13). One of the most recent frontiers of CA therapy concerns orthodontic treatment in growing patients (14–16).

Some authors have reported significative improvements in maxillary arch width of growing patients treated with CA (17–22), suggesting that CA could be a reasonable alternative to traditional slow maxillary expanders (18). Furthermore, CA with mandibular advancement (MA) systems have been found to be effective in treating Class II growing patient with a retrognathic mandible (23–29). Therefore, the aim of the present study was to systematically search the literature and summarize the current available scientific evidence regarding the effect of CA treatment in children and adolescent patients, and evaluate the advantages of aligners treatment compared to traditional appliances in term of dentoskeletal effects, periodontal health and quality of life.

## 2 Materials and methods

### 2.1 Protocol and registration

The study protocol was established and registered in the OSF registries (https://osf.io/wmerq).

The current systematic review was conducted in accordance to the PRISMA guideline (30). The review question was structured based on the PICO approach (31):

P (patients): humans, both males and females, less than 18 years of age (children and adolescents),
I (intervention): orthodontic treatment with CA,
C (comparison): other orthodontic treatments, no treatment or no comparison,
O (outcome): dentoalveolar and skeletal effects (primary outcome); adverse effects, periodontal effects, compliance, quality of life, aesthetics (secondary outcome).

### 2.2 Literature search and study selection

An electronic search without time or language restrictions was performed in December 2021 on the following electronic databases: PubMed, Scopus, The Cochrane Library and Literature in the Health Sciences in Latin America and the Caribbean (LILACS), as shown in Table 1. A manual search was also performed among the references of the included articles to identify possible items not listed in the electronic search. The following inclusion criteria were used for the study selection: human study in children and adolescent patients (<18 years of age); studies with at least one group of treated patients (CA treatment). Randomized clinical trial (RCT), prospective and retrospective non-randomized clinical trials, and studies without control group were included. Case series and case reports (<10 patients), *in vitro*/laboratory studies, systematic review, narrative reviews, editorials, opinion articles or letter from authors, were excluded. Two authors (RB and VDS) independently screened the list of title and abstract of potentially eligible studies, using the Rayyan (http://rayyan.qcri.org) software (32). If the title and abstract did not provide sufficient information, or if the abstract was not available, articles were included for full-text assessment. Disagreements between the two investigators were resolved through discussion and if necessary, a third operator (VD) was contacted for final decision.

**Table 1:** Search strategy for each database and relative results.

Database	Search strategy	Results
PubMed www.ncbi.nlm.nih.gov	((((((((“Clear aligner appliance” [tw]) OR (“Invisalign First” [tw])) OR (“Invisalign” [tw])) OR (“Clear Aligner” [tw])) OR (“Aligner” [tiab])) OR (“Clear aligner therapy” [tw])) OR (“Invisible Orthodontic” [tw])) OR (“Transparent aligner” [tw])) AND (((((((((((“mixed dentition” [tiab]) OR (“child” [tiab])) OR (“teen” [tiab])) OR (“adolescent” [tiab])) OR (“growing” [tiab])) OR (“early treatment” [tiab])) OR (“expansion” [tiab])) OR (interceptive orthodontic [tiab])) OR (functional orthodontic [tiab])) OR (functional orthopaedic [tiab])) OR (functional orthopedic [tiab]))	192
Scopus http://www.scopus.com/	(TITLE-ABS-KEY (clear AND aligner) OR TITLE-ABS-KEY (invisalign) OR TITLE-ABS-KEY (invisible AND orthodontic) OR TITLE-ABS-KEY (transparent AND aligner)) AND (TITLE-ABS-KEY (mixed AND dentition) OR TITLE-ABS-KEY (growing) OR TITLE-ABS-KEY (child) OR TITLE-ABS-KEY (teen) OR TITLE-ABS-KEY (adolescent) OR TITLE-ABS-KEY (early AND treatment) OR TITLE-ABS-KEY (interceptive AND orthodontic) OR TITLE-ABS-KEY (functional AND orthodontic) OR TITLE-ABS-KEY (functional AND orthopeadic) OR TITLE-ABS-KEY (functional AND orthopedic))	287
Cochrane Library www.cochranelibrary.com	(clear aligner):ti,ab,kw OR (invisalign):ti,ab,kw OR (transparent aligner):ti,ab,kw OR (invisible orthodontic):ti,ab,kw AND (“mixed dentition”):ti,ab,kw OR (“Child”):ti,ab,kw OR (teen):ti,ab,kw OR (adolescent):ti,ab,kw OR (growing):ti,ab,kw OR (“functional orthodontic therapy”):ti,ab,kw OR (“interceptive orthodontics”):ti,ab,kw OR (early treatment):ti,ab,kw	34
Latin American and Caribbean Health Sciences (LILACS) http://lilacs.bvsalud.org	((“clear aligner”) OR (“transparent aligner”) OR (“invisible orthodontic”) OR (invisalign)) AND ((mixed dentition) OR (growing) OR (children) OR (child) OR (teen) OR (teenager) OR (adolescent) OR (interceptive) OR (functional)) AND (db:(“LILACS” OR “BBO”))	137

### 2.3 Data extraction

Data were independently extracted by two authors (RB and VDS) using a customized extraction form. The authors were not contacted for further details. The following data were extracted: author; year and country of publication; study design (RCT, CCT, Ret etc.) and sample size; baseline orthodontic diagnosis; presence of control group; appliance; wearing time; aligners change (days); mean number of aligners; dropout; follow-up; methods of measurement; study aim; outcome; and author's conclusions.

### 2.4 Methodological quality of the included studies

To evaluate the risk of bias of randomized controlled trials (RCT), the Cochrane Collaboration “risk of bias” (RoB-2) tool was used (33). Risk of bias was assessed and judged as low risk, high risk, or unclear risk of bias for seven domains.

For non-randomized studies, the Cochrane Collaboration “risk of bias in non- randomized studies of interventions” (ROBINS-I) tool was applied (34), and studies were rated as low, moderate, serious or critical risk of bias.

## 3 Results

### 3.1 Search results

**Figure 1:**
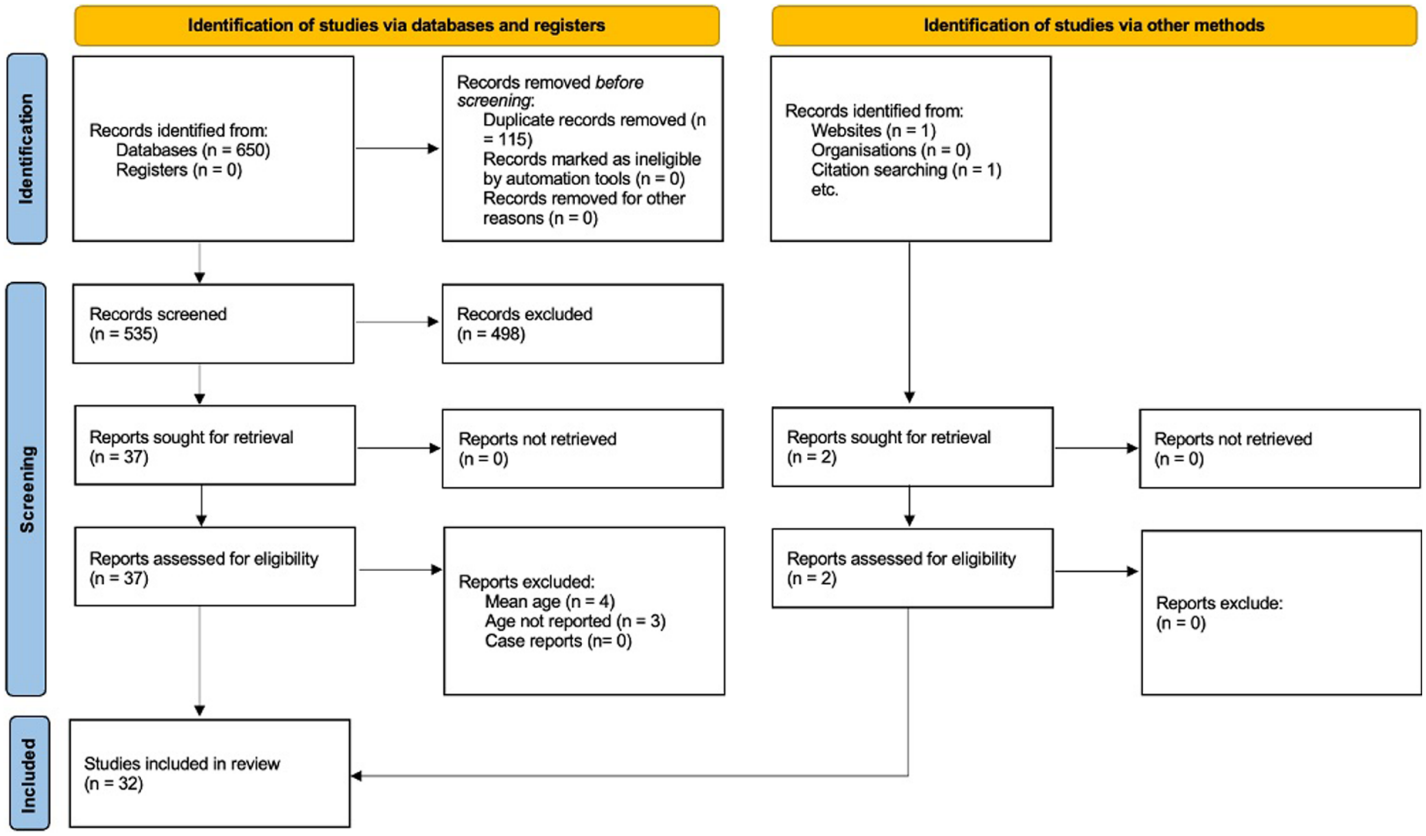
PRISMA flow ndiagram of the included studies.

The PRISMA flow chart describing the study identification process is presented in Figure 1.

A total of 650 records were identified through electronic and manual searches. After duplicates removal, title and abstracts of 535 records were screened, of which 37 met the inclusion criteria and were considered as potentially eligible. After full-text reading, seven studies were excluded, with the most common reason for exclusion being the age of the study sample (Table 2). One article was retrieved from sources other than database (25), while another study was found through additional hand-searching of the reference lists of selected studies (29). Thus, 32 articles were finally included in this systematic literature review (4, 5, 11, 14, 17–21, 23–29, 35–50).

**Table 2:** List of full text excluded and reason for exclusion.

Author, year	Title	Reason for exclusion
Deregibus, 2020	Morphometric analysis of dental arch form changes in class II patients treated with clear aligners	No information about patients’ age
Graciela, 2020	Expansion treatment using Invisalign®: Periodontal health status and maxillary buccal bone changes. A clinical and tomographic evaluation.	Adult patients
Lanteri, 2018	The efficacy of orthodontic treatments for anterior crowding with Invisalign compared with fixed appliances using the Peer Assessment Rating Index	Adult patients
Meazzini, 2020	Comparison of pain perception in patients affected by cleft and cranio Facial Anomalies treated with traditional fixed appliances or Invisalign	Adult patients
Meazzini, 2020	Comparison of the psychosocial impact on patients affected by cranio facial anomalies between traditional orthodontic brackets and aligners	Adult patients
Vidal-Bernárdez, 2021	Efficacy and predictability of maxillary and mandibular expansion with the Invisalign® system	No information patients’ age
Inchingolo. 2023	Predictability and Effectiveness of Nuvola® Aligners in Dentoalveolar Transverse Changes: A Retrospective Study	No information about patients’ age

### 3.2 Characteristics of the studies

Data extracted from the 32 studies are presented in Table 3. The studies included in the present review were conducted between 2015 and 2023. The number of total subjects included in each review ranged between 15 and 113, and the median age was 8.7 years (IQR 7.6–9.8 years).

**Table 3:** Data extracted from the 32 included studies.

Author year country	Study design Sample (n. males females; Age)	Baseline orthodontic diagnosis	Group (G)/Control (C) ©	Appliance	Wearing time	Aligner change	Mean n. of aligners	Dropout	Follow-up	Study material	Study aim	Outcome	General conclusions
Abbate et al., 2015 Italy (11)	Preliminary RCT 50 (aged 10–18 years) G1 = 25 G2 = 25	NR	G1: CA G2: FMB	G1: Invisalign® aligners	20–22 h per day and removed only for eating and tooth brushing	every 2 weeks	NR	G1 = 3	pre-treatment (T0) after 3 months (T3) after 6 months (T6) after 12 months (T12)	Microbiological analyses Clinical assessment	Microbiological and periodontal changes	PD PI BOP. Compliance with oral hygiene procedures FMPS FMBS	None of the patients was positive for the periodontal anaerobes analyzed. The PI PD BOP FMPS and FMBS scores were significantly lower and compliance with oral hygiene was significantly higher in CA group than in FMB group.
Bahammam et al., 2023 Canada (48)	Retrospective 22 (16 F 6 M) G1 = 11 mean age 16.27 ± 0.56 years G2 = 11 mean age 15.5 ± 1.53 years	trasverse maxillary discrepancy	G1: CA G2: Quad Helix	G1: Invisalign® aligners G2: Wilson-type quad helix	NR	every 7 days	NR	NR	pre- expansion (T0) and after expansion (T1) mean treatment time 1.6 ± 0.4 years	CBCT	Maxillary alveolar bone thickness and height changes	left and right canines premolars and molars bone height and thickness	Decrease in buccal alveolar bone thickness and heights in patients treated by quad helix compared to CA
Blackham et al., 2020 Columbia (29)	Retrospective 64 G1: 32 mean age 13.15 ± 1.37 years G2: 32 mean age 11.82 ± 1.74 years C1: 32 mean age 13.07 ± 1.35 years	Dentoskeletal Class II	G1: CA + MA G2: FA	G1: Invisalign® aligners G2: TB	NR	NR	NR	G1 (T3) = 13 G2 (T3) = 13	pre-treatment (T1) post-advancement mean treatment time 356 days (T2) post treatment (T3)	Lateral ceph	Skeletal dental and soft tissue changes	SNB° ANB° overjet L1-MP lower incisor protrusion (L1-NB mm) Facial Convexity (soft tissue) overbite U1 proclination and protrusion mandibular length skeletal convexity molar positioning	Both CA + MA and TB are effective in correcting a Class II malocclusion. Treatment with CA + MA may result in less proclination of the lower incisors compared to the TB appliance.
Borda et al., 2020 USA (4)	Retrospective 52 (from 11 to 17 years) G1 = 26 (mean age 13.7 ± 1.4 years) G2 = 26 (mean age 13.0 ± 1.3 years)	“mild” malocclusion (ABO Discrepancy index)	G1: CA G2: FMB	G1: Invisalign ® aligners G2: fixed appliance	NR	NR	NR	NR	pre-treatment (T0) post-treatment (T1) * * treatment duration NR	pre-treatment: digital models lateral ceph; post-treatment: digital models panoramic radiographs. Chart reviews	Dental effects and efficiency	Alignment occlusal relation OVJ marginal ridges buccolingual inclination occlusal contacts interproximal contacts root angulation. N. of appointments n. of emergency visits and overall treatment time	Equivalent effectiveness of CA compared to FMB with significantly improved results for CA treatment in terms of tooth alignment occlusal relations and overjet. Assessment of the n. of appointments n. of emergency visits and overall treatment time showed better outcomes for treatment with CA.
Caruso et al., 2021 Italy (24)	Retrospective 20 (10 M 10 F; mean age 10 ± 1.03 years) G1 = 10 (mean age 10 ± 1.05 years) G2 = 10 (mean age 10 ± 1.05 years)	SNB < 78 and ANB > 4	G1: FA G2: CA + MA	G1: TB G2: Invisalign® aligners	NR	NR	NR	NR	pre-treatment (T0) post-treatment (T1) * * treatment duration NR	Lateral ceph	Dentoskeletal effects	SNA° SNB° ANB° OVJ OVB Go-Me ^ANSPNS° Ar-Go ^ Go-Me° FMA° U1 ^ ANSPNS° L1 ^ GoMe°	The present data show the effectiveness of both TB and CA + MA in the management of skeletal Class II malocclusions due to mandibular retrusion. CA + MA seems indicated in Class II cases where a control of the upper frontal teeth position is needed.
Chhibber et al., 2017 USA (35)	RCT 71 (41 M 30 F; mean age 15.6 years) G1 = 27; G2 = 22; G3 = 22	<5 mm of anterior crowding or spacing with adequate OVJ and OVB	G1: CA G2: SLB G3: ELB	G1: Invisalign ® aligners G2: Carriere G3: Ortho Organizers Inc	20 h per day	every 2/3 weeks	NR	G1 = 3 G2 = 5 G3 = 2	pre-treatment (T0) after 9 months (T1) after 18 months (T2)	Clinical assessment	Periodontal changes	PI GI PBI	No evidence of any significant differences in oral hygiene levels among CA SLB and conventional ELB after 18 months of active orthodontic treatment.
Chou et al., 2023 USA (42)	Retrospective 72 (12–18 years) G1 = 47 (27 M 20 F mean age 13 ± 2 years) G2 = 25 (11 M 13 F mean age 13 ± 1 years)	Class I and II moderate to severe malocclusions	G1: CA G2: FMB	G1: Invisalign® aligners G2: Damon system	NR	NR	NR	NR	before (T0) and after (T1) treatment; mean treatment time G1: 24 months G2: 27 months	Digital models Lateral ceph	Efficacy and efficiency	DI and CRE treatment duration n. of scheduled visits and n. of emergency visits	CA vs FMB cases were completed 3 months faster with eight fewer visits but treatment efficacy was not significantly different. Greater lower incisor proclination in the FMB group compared to the CA group.
Cremonini et al., 2022 Italy (26)	Retrospective 15 (7 F 8 M mean age 10.3 years)	SNB <78° ANB >4° full class II or head-to-head molar relationship OVJ < 10 mm FMA <27° CSV3-CSV4	G1: CA + MA and Class II elastics	G1: F22® Young	15/20 h per day Class II elastics during night	No aligners change	One upper advancement aligner associated with a lower prescription aligner	NR	pre-treatment (T0); post-treatment (T1); mean treatment time 10 months ± 0.5	Digital models Lateral ceph	Dentoskeletal effects	SNA SNB ANB Wits FMA MP-SN U1-Occl plane U1-Palatal Plane L1-Occl Plane IMPA SN-PP PP-GoGn Go-Pg Co-Gn OVJ OVB Molar Class	Significant increase in the total mandibular length forward shift of point B normalization of the sagittal relationship between the jaws. A dental compensation has to be taken into consideration because a proclination of lower incisor and extrusion of molars.
Cretella Lombardo et al., 2022 Italy (45)	Retrospective 32 G1 = 17 (8 M 9 F; mean age 8.1 ± 0.8 years) G2 = 15 (7 M 8 F; mean age 8.4 ± 1.1 years)	Posterior transversal discrepancy up to 6 mm mesial step or flush terminal plane molar relationship	G1: RME G2: CA	G1: butterfly palatal expander G2: Invisalign® First system	full time	every 7 days	NR	NR	pre-treatment (T1); post-treatment (T2); mean treatment time 8 months	Digital models	Maxillary morphological changes	Maxilary arch form	CA treatment can induce significant morphological modifications of the upper arch shape compared to RME therapy. At the end of the treatment the CA subjects presented an improvement in the maxillary arch shape differently from the RME subjects who maintained the initial triangular shape.
Cretella Lombardo et al., 2023 Italy (28)	Retrospective 71 children G1 = 35 (17 M 18 F; mean age 12.0 ± 1.3 years) G2 = 21 (9 M 12 F; mean age 11.2 ± 1.1 years) C1: 15 (4 M 11 F; mean age 10.9 ± 1.1 years)	5 < OVJ < 8 mm bilateral full class II or end-to-end molar relationships ANB > 4° CVM3	G1: FA G2: CA + MA C1: untreated	G1: TB G2: Invisalign® aligners	full time except when eating drinking or brushing	every 7 days	NR	NR	pre-treatment (T0) post-treatment (T1) * * treatment duration is not reported	Lateral ceph	Dentoskeletal effects	SNA° SNB° ANB° Wits Co-Gn TVL-Pg SN-Pal.Pl. SN- Mand. Pl.° Pal. Pl.- Mand. Pl.° CoGoMe° OVJ OVBUpper Inc.-Pal. Pl.° Lower Inc.-Mand. Pl.°	Treatment with the CA + MA and TB appliances produced a significant elongation of the mandible with an improvement in sagittal relationship OVJ and OVB and with good control of the vertical relationship. TB subjects showed a greater advancement of the soft tissue chin.
Cretella Lombardo et al., 2023 Italy (19)	Retrospective 32 G1 = 17 (8 M 9 F; mean age 8.1 ± 0.8 years) G2 = 15 (7 M 8 F; mean age 8.4 ± 1.1 years)	Posterior transversal discrepancy up to 6 mm mesial step or flush terminal plane molar relationship	G1: RME G2: CA	G1: butterfly palatal expander G2: Invisalign® First system	full time	every 7 days	NR	NR	pre-treatment (T1); post-treatment (T2); mean treatment time 8 months	Digital models	Dental effects	III-III IV-IV V-V 6-6 mesial cusps 6-6 distal cusps 6–6 transpalatal	RME widened the palate tipping the first upper molars buccally to a greater extent whereas the CA caused a greater increase in the canine width.
da Silva et al., 2023 Brazil (44)	RCT 32 G1 = 14 (6 F 8 M mean age: 9.33 years) G2 = 13 (9 F 4 M mean age: 9.65 years)	Little's Irregularity Index in the maxillary arch of at least 3 mm.	G1: CA G2: FMB	G1: thermoplastic aligners G2: Preadjusted brackets (2 × 4)	20 h per day	every 2 weeks	10 aligners in the treatment phase and 6 aligners in the refinement phase	G1 = 2 G2 = 3	pre-treatment (T0) at the end of the treatment (T1)	Digital models clinical assessment	Dental effects and efficiency	maxillary incisor irregularity index treatment time arch width arch perimeter arch length arch size arch shape incisor leveling incisor mesiodistal angulation PI white spot lesion	Clear aligners and fixed 2 × 4 mechanics showed similar efficacy and efficiency for the correction of maxillary incisor crowding in the mixed dentition. Both appliances showed similar dental PI and white spot lesion incidence during treatment.
Dianiskova et al., 2022 Italy (14)	Retrospective 49 (32 F 17 M mean age ± SD 12.9 ± 1.7 years) G1 = 25 (16 F 9 M mean age ± SD 13.1 ± 1.8 years) G2 = 24 (16 F 8 M mean age ± SD 12.7 ± 1.7 years)	Class II division 1 ANPg ≥ 3° OVJ ≥ 4 mm at least End-to-End Class II molar and canine relationship molar and canine Class I at the end of the treatment	G1: FMB G2: CA	G1: MBT prescription G2: Invisalign® aligners	full time	every 7 days	NR	NR	pre-treatment (T0) post-treatment (T1) * * treatment duration is not reported	Digital models Lateral ceph	Dentoskeletal effects	SNA° SNPg° ANPg° Wits Co-Gn SN/PP° SN/MP° PP/MP° CoGoMe° Co-Go° Co-Go OVJ OVB U1/PP° L1/GoGn°	Class II elastics combined with CA and FMB produce a similar correction on ANPg° in growing patients. CA presented a better control of L1/GoGn°. CA and elastics might be a good alternative in the correction of mild Class II malocclusion in cases where a proclination of lower incisors is unwanted.
Dianiskova et al., 2023 Italy (41)	Cross-sectional study 56 (19 F 37 M mean age 10 years) G1 = 28 (7 F 21 M mean age 11 years); G2 = 28 (12 F 16 M mean age 9 years)	NR	G1: elastodontic appliances G2: CA	NR	NR	NR	NR	NR	post-treatment	questionnaires	Patients' and parents' satisfaction	Self-made questionnaire	According to the parents school life and social life were significantly improved in the CA group as compared to the Elastodontic appliance group. Parents belonging to the CA group found that their child's treatment was much shorter than expected.
Favero et al., 2023 Italy (43)	Prospective 43 (27 F mean age 15.25 ± 1.65 years and 16 M mean age 15.65 ± 2.36 years)	Angle Class I mild or medium crowding in the upper arch	G1: CA with a vestibular rim (VR) G1: CA with juxtagingival rim (JR)	G1: Scheu Dental	NR	3 months	Two experimental aligners with different edge design	G1 = 5	pre-treatment (T0) after 3 months (T1) after further 3 months (T2)	Clinical examination	Periodontal effects	PI GI GBI	Worsened inflammatory indices with JR. VR had a protective effect reducing the risk of mechanical trauma.
Gonçalves et al., 2023 Portugal (47)	Retrospective 24 (11 M 13 F) between 6 and 12 years	patients requiring maxillary expansion	G1: CA	G1: Invisalign® First system	at least 22 h per day	every 7 days	NR	NR	Mean treatment time 18 months	Digital models	Transveral changes and predictability	Mesiopalatal cusp tip of the temporary and permanent molars palatal cusp tip of the premolars cusp tip of temporary and permanent canines	Mean maxillary expansion 6.0 mm with an efficiency of 62.6 ± 18.3%. Mean mandibular expansion 3.5 mm with an expansion efficiency of 61.6 ± 32.1%.
Kong et al., 2023 China (27)	Retrospective 30 (15 F 15 M mean age 11.6 ± 0.9 years)	between CVMS1 and CVMS3; SNB ≤ 78° mixed dentition ANB ≥ 6° permanent dentition ANB ≥ 5°; SN/MP ≤ 37°	G1: CA + MA	G1: Invisalign® aligners	NR	NR	NR	NR	pre-treatment (T0) post-treatment (T1) * * treatment duration is not reported	Lateral ceph	Dentoskeletal effects	ss/OLP Pg/OLP Co/OLP Pg/OLP + Co/OLP Co-Go Go-Pg Co-Pg SNA SNB ANB Wits SN-MP S-Go/N-Me ANS-Me/N-Me	CA + MA can effectively promote the growth development and appearance of the mandible. The treatment effect has both dental and skeletal effects with skeletal effects having a stronger influence.
Levrini et al., 2021 Italy (18)	Retrospective 20 (12 F 8 M mean age 8.9 years)	Mild crowding or limited transverse maxillary deficiency	G1: CA	Invisalign® First system	NR	The 1st aligner for 14 days and then weekly change	33	NR	pre-treatment (T0) post-treatment (T1); mean treatment time 8 months	Digital models	Trasverse maxillary changes	Canine gingival width. second deciduous molar gingival width P6 gingival width First deciduous molar dental width Second deciduous molar dental width First permanent molar dental width Arch perimeter Arch depth Intermolar angle	CA demonstrate increased arch width
Lione et al., 2021 Italy (17)	Prospective 23 (9 F 14 M mean age 9.4 ± 1.2 years)	posterior transverse discrepancy arches up to 6mm	G1: CA	G1: Invisalign® First system	full time except during meals and tooth brushing	every 7 days	37 upper and 37 lower	NR	pre-treatment (T1) after 9 months (T2)	Digital models	Transverse maxillary changes	III–III IV–IV V–V 6–6 mesial cusps 6–6 distal cusps 6–6 transpalatal	CA can be considered effective in maxillary arch development. The greatest net increase was detected at the level of upper first deciduous molars whereas the upper first molars showed a greater expansion in the intermolar mesial width due to a rotation that occurs around its palatal root.
Lione et al., 2022 Italy (46)	Prospective 36 (16 M 20 F 9.9 ± 1.9 years)	Molar Class II edge-to-edge	G1: CA	G1: Invisalign® First system	full time except during meals and tooth brushing	every 7 days	32 upper aligners	NR	pre-treatment (T0); post-treatment (T1); mean treatment time 8.6 months	Digital models	Dental effects trasverse maxillary changes and predictability	Henry's angle Mesiobuccal Expansion Distobuccal Expansion Mesiobuccal Sagittal Distobuccal Sagittal	CA effectively produces an arch expansion and upper molars' distal rotation. Upper molar derotation provides a 1 mm of gain in arch perimeter and occlusal improvement.
Lione et al., 2022 Italy (40)	Prospective 18 (10 F 8 M; mean age 9.4 ± 1.2 years)	dento-alveolar transverse discrepancy of 3–6 mm mild/moderate crowding mesial step or a flush terminal plane molar relationship SN^GoGn angle from 27° to 37°	G1: CA	G1: Invisalign® First system	full time except when eating drinking or brushing	every 7 days	NR	NR	pre-treatment (T0) after the first set of aligners (T1)	Intraoral photograph digital models	Gingival margins' modifications	Gingival margin height deciduous canine inclination crown length	Sequential expansion protocol and correction of anterior crowding induced significant modifications of gingival contour resulting in a more harmonious smile. Specifically these modifications are represented by reduced gingival margin height of upper permanent incisors upper deciduous canine and molars and increased upper deciduous canine inclination.
Lu et al., 2023 China (20)	Prospective 51 (6–10 years) G1 = 17 G2 = 17 C1 = 17	posterior transverse discrepancy ≤ 5 mm; mild or moderate crowding; CS1–CS3 in cervical vertebral maturation	G1: CA G2: RME C1: untreated	G1: Invisalign® First system G2: acrylic splint expander	all day except for meals and tooth brushing	every 7 days	NR	NR	pre-treatment (T0) after 6 months (T1)	Digital models	Dental effects	Intercanine/Intermolar dentoalveolar width Arch depth Arch perimeter Inclination of the molars	Both CA and RME can expand the maxillary arch in mixed dentition. RME shows significant better efficiency of dental arch expansion than CA
Ravera et al., 2021 Italy (23)	Prospective 72 (8–15 years) G1 = 40; C = 32 CVM2 study group = 20 CVM3 study group = 20 CVM2 control group = 15 CVM3 control group = 17	skeletal Class II with mandibular retrusion (3°<ANB < 8°); normal divergence (SN^GoGn < 37°); moderate crowding in the upper arch (≤4 mm)	G1: CA + MA C1: untreated	G1: Invisalign® aligners MA	NR	NR	NR	G1 = 4 C = 4	pre-treatment (T0); post-treatment (T1); mean treatment time 18 months	Lateral ceph	Dentoskeletal effects	SN^GoGn SpP^GoGn SNA SNB ANB; A-Pog Wits Go-Gn Co-Go Co-Gn; X11-SpP X41-GoGn X11-X41	The use of CA + MA is effective in treating Class II growing patient with retrognathic mandible in the short-term period. While treatment at prepubertal stage of growth results in dentoalveolar rather than skeletal effects treatment during the pubertal spurt produces skeletal effects with an annual rate of change of 5.8 mm.
Sabouni et al., 2022 UAE (51)	Retrospective 32 (13 F 19 M mean age 13 years (range 9.9–14.8 years)	Class II	G1: CA + MA	G1: Invisalign® aligners MA	NR	every 7 days	37 (30–55)	NR	pre-treatment (T1); post-treatment (T2); mean treatment time 9 months	Lateral ceph	Dentoskeletal and soft tissue effects	SNA SNB ANB Wits convexity mandibular length MP-SN FMA U1-L1 IMPA OVJ OVB Soft tissue nasolabial angle and the chin angle	CA + MA took approximately 9 months for 1.5 mm of overjet correction. The lower incisor angulation was maintained during class II correction. There were only minor skeletal changes in favor of class II correction.
Sauer et al., 2022 Germany (39)	Case-control 40 (18 F 22 M mean age 13.6 years range 11–17 years)	NR	G1: CA	G1: Invisalign® Teen system	NR	NR	NR	NR	start of therapy (T1); after 4 weeks (T2); after 10 weeks (T3); after 6 months (T4); after 1 year (T5)	Questionnaire	Oral health-related quality of life and oral hygiene	OHIP-G1 PIDAQ TMQH	No increased dental plaque accumulation. Minor restrictions in the quality of life and increased psychosocial well-being.
Sharma et al., 2021 Canada (38)	Cross-sectional case-control 74 (30 M 44 F mean age 14.9 ± 1.9 years range: 11–18 years) G1 = 37; G2 = 37	IOTN Grade 2 or 3	G1: FMB G2: CA	G2: Invisalign® aligners	NR	NR	NR	NR	after a minimum of 6 months of treatment	Questionnaires	Treatment impacts quality of life and satisfaction	COHIP + additional questions	Both treatment groups were generally very satisfied with their treatment modality. The overall quality of was similar in FMB and CA.
Shen et al., 2021 China (36)	Retrospective 113 * G1 = 56; G2 = 57 *age is not reported	malocclusion* *characteristics not reported	G1: FMB G2: CA	G2: Invisalign® aligners	NR	every 2 weeks	NR	NR	pre-treatment (T0) post-treatment (T1) * * treatment duration is not reported	Clinical assessment microbiological analysis questionnaire	Dental effects periodontal changes quality of life patient satisfaction adverse reactions	Overbite alignment satisfaction PI PBI PD GI TNF-α IL-6 IL-2 levels occurrence of adverse reactions COHIP + additional questions	The efficacy of CA treatment in children with malocclusions is higher than it is using traditional FMB as it helps improve their chewing function periodontal health and quality of life and helps reduce the inflammatory factor levels and improves their satisfaction with the orthodontic treatment.
Sifakakis et al., 2018 Greece (37)	Prospective 30 (12–18 years) G1 = 15; G2 = 15	NR	G1: FMB G2: CA	G1: self-ligating bracket G2: passive aligners	full time except when eating drinking or brushing their teeth.	after 2 weeks	2	NR	baseline (T0) after 2 weeks (T1) after 1 month (T2)	Clinical assessment microbiological analysis	Microbiological and periodontal changes	PI GI DMFT index salivary cariogenic bacteria	There were no differences in the salivary counts of S. mutans or L. acidophilus among adolescent patients treated for 1 month with CA or self-ligating appliances. On the other hand patients treated with CA had lower salivary levels of S. sanguinis compared to those treated with FMB.
Sun et al., 2022 China (25)	Retrospective 46 (23 F 23 M mean age 13.66 ± 4.25 years) G1: 23 (11 F 12 M mean age 15.25 ± 4.93 years) G2: 23 (12 F 11 M mean age 12.07 ± 2.63 years)	Class II Division 1 mandibular retrognathia at least an end-to-end molar relationship; Overjet between 5 and 10 mm; SNB <78° and ANB >5°	G1: FA G2: CA	G1: TB G2: Angel Aligner A6 MA Solution	for at least 17 h/day	NR	NR	NR	pre-treatment (T0); post-treatment (T1); mean treatment time G1: 9.40 ± 4.23 months G2: 10.23 ± 3.27 months	Laterap ceph	Dentoskeletal and soft tissue effects	SNA SNB ANB GoGn-SN Co-A Co-Gn Go-Me Go-Gn N-Me ANS-Me S-Go N-S-Ar S-Ar-Go Ar-Go-Me NA-Pog Pog-NB U1-SN U1-PP U1-NA U6-PP IMPA L1-NB L1-MP L6-MP U1-L1 OVJ OVB UL-E-Line LL-E-Line Z-angle H-angle nasolabial angle	Both CA and TB can correct Class II malocclusion retract the upper anterior teeth tilt the lower anterior teeth coordinate the differences between the maxilla and mandible. CA has more advantages in adduction of anterior teeth and backward movement of point A while TB has more advantages in forward movement of point B. Both kinds of appliances can lead to an increase in the proportion of lower 1/3 of the face.
Wang et al., 2023 Canada (21)	Retrospective 63 (8–11 years) G1 = 23 G2 = 23 C1 = 23	mixed dentition malocclusions	G1: CA G2: SME C1: untreated	G1: Invisalign® First system G2: Haas-Expander	NR	every 7 days	28	NR	before (T0) and after (T1) treatment; mean treatment time G1: 1.02 ± 0.36 years G2: 0.98 ± 0.51 years Mean observational period C1: 1.22 ± 0.56 years	Digital models	Trasverse maxillary changes	Intercanine width intermolar width palatal surface area and volume first molar buccolingual inclinations	CA produced significant increases iniIntercanine and intermolar width compared to untreated C. However CA expansion magnitude was less than that in the SME group. The overall palatal SA and volume changes after CA treatment showed no significant differences compared to the CG while the SME group showed a significant increase in palatal dimensions. Molar inclinations were unchanged after CA but SME increased Molar Inclination significantly.
Wu et al., 2023 China (50)	Retrospective 63 (37 M 26 F) G1 = 14 (7 F 7 M mean age 10.71 ± 1.44 years) G2 = 11 (7 F 4 M mean age: 11.55 ± 0.69 years) G3: 12 (5 F 7 M mean age: 11.55 ± 0.69 year) G4: 14 (2 F 12 M mean age of 12.11 ± 1.16 years) C1: 12 (5 F 7 M mean age: 10.41 ± 0.90 year)	skeletal class II with ANB >4°; overjet > 5 mm; Angle class II molar and canine relationship; CVM2	G1: FA G2: FFA G3: FA G4: CA + MA C1: untreated	G1: Van beek activator G2: Herbst appliance G3: TB G4: Invisalign® aligners + MA	NR	NR	NR	NR	pre-treatment (T1); post-treatment (T2) Mean treatment time: G1: 7.28 ± 2.30 G2: 10.18 ± 3.06 G3: 10.16 ± 5.46 G4: 22.84 ± 8.98 C1: 10.25 ± 3.74	Lateral ceph	Dentoskeletal effects	SNA SNB ANB FH-NP NA-PA MP-FH MP-SN Co-Go Go-Pog Co-Pog Y Axis Angle Lower Facial Height Ratio Vertical Ratio P-A Face Height U1-SN U1-PP U6-PP L1-MP U1-L1 OP-FH	Four appliances are all effective in mandibular advancement modification of class II molar relationship and overjet with increase in lower facial ratio. Vanbeek Activator has the most skeletal effects. Vanbeek and MA have a good control of mandibular incisors while more compensatory lower incisors proclination in Herbst and TB. Herbst has greater maxillary molar distalization. MA allows aligning and leveling meanwhile leading the mandible forward.
Zybutz et al., 2021 Canada (5)	Survey study 68 G1 = 45 (18 M 27 F mean age 13.62 ± 1.54 years); G2 = 23 (13 M 10 F mean age 10.60 ± 1.92 years)	NR	G1: CA + MA G2: FA	G1: Invisalign® aligners + MA G2: TB	NR	NR	NR	NR	pre-treatment (T0) after at least 2 months (T1)	questionnaires	Patients' experiences	social and functional changes	TB and CA patients shared similar experiences for most of the parameters measured but there were significant differences between the groups regarding appliance wear and management discomfort and function.

6-6 distal cusps, First intermolar distal width; 6-6 mesial cusps, First intermolar mesial width; 6-6 transpalatal, First intermolar transpalatal width; BOP, bleeding on probing; CA, clear aligners; COHIP, child oral health impact profile; DMFT, decayed, missing, and filled teeth; ELB, preadjusted edgewise fixed appliance with elastomeric ligated brackets; FA, functional appliance; FFA, fixed functional appliance; FMB, multibrackets fixed appliance; FMBS, M3 full mouth bleeding score; FMPS, full mouth plaque score; GI, gingival index; III-III, Intercanine width; IL-2, interleukin-2; IL-6, interleukin-6; IV-IV, first interdeciduous molar width; MA, mandibular advancement; NR, not reported; PBI, periodontal bleeding index; PD, probing depth; PI, plaque index; SLB, preadjusted edgewise fixed appliance with self-ligated brackets; TB, twin-block; TNF-α, the tumor necrosis factor-α; V-V, Second interdeciduous molar width.

Baseline orthodontic diagnoses were transverse maxillary deficiency (seven studies), crowding (three studies), and Class II (12 studies); however, not all of the studies clearly specified the initial diagnosis. Three studies included patients with different degree of malocclusion (according to standardized indices such as the ABO malocclusion index, IOTN and Little's Irregularity index), while seven studies did not mention the baseline malocclusion as an inclusion criterion.

Of the 32 included studies, three were RCTs, and 29 were non-randomized studies (18 retrospective, eight prospective, and three cross-sectional studies). A comparison with fixed orthodontic appliance was performed in nine studies (4, 11, 14, 35–38, 42, 44), two of which adopted self-ligating appliances (35, 37). Five studies compared the effects of CA and mandibular advancement (MA) with those of the Twin-Block appliance (TB) (5, 24, 25, 28, 29), or other functional appliances (50). Four studies compared the effects of CA with rapid maxillary expander (RME) with different designs (19–21, 45), or Quad Helix (48). Only five studies included a sample of untreated subjects as a control group (20, 21, 23, 28, 50), and eight studies had no control group (17, 18, 27, 39, 40, 46, 47, 49). Concerning the treatment protocol for CA use, five studies recommended aligners change every 2/3 weeks (11, 35–37, 44). 12 studies recommended a weekly change of the aligners (14, 17, 19–21, 29, 40, 45–48, 52). One study proposed the use of the first pairs of aligners for two weeks, and then the change every seven days (18). In the study conducted by Favero et al. (43), two experimental aligners with different edge design were used in the same treatment group, with the device change occurring after three months (33). In one study there was no change in aligners, as only one upper advancement aligner associated with a lower prescription aligner was used during treatment (26). Furthermore, in another study, only two pairs of passive experimental CA were adopted, with the first pair being changed after two weeks (37). Twelve studies did not mention a specific wearing time protocol, suggesting adherence to the manufacturer's recommendations (4, 5, 23–25, 27, 29, 38, 39, 41, 42, 50). The mean number of aligners required to complete the therapy was reported only in six studies, ranging between 28 and 37 (17, 18, 21, 44, 46, 52). The follow-up data ranged from two weeks up to 24 months (7, 12, 17–22, 24, 37–39, 41–48, 50, 52, 53).

### 3.3 Risk of bias (quality assessment) of the included studies

#### 3.3.1 RCT

The three RCTs (11, 35, 44) were judged to be at an overall low risk of bias (Table 4).

**Table 4:** Risk of bias in randomized trials (RoB 2 tool).

First author, year	Bias arising from the randomization process	Bias due to deviations from intended interventions	Bias due to missing outcome data	Bias in measurement of the outcome	Bias in selection of the reported result	Overall risk of bias
Abbate et al., 2015 (11)	Low risk	Low risk	Low risk	Low risk	Low risk	Low risk
Chhibber et al., 2017 (35)	Low risk	Low risk	Low risk	Low risk	Low risk	Low risk
da Silva et al., 2023 (44)	Low risk	Low risk	Low risk	Low risk	Low risk	Low risk

#### 3.3.2 Non-randomized studies

Out of the seven prospective studies (17, 20, 22, 37, 40, 43, 46), two were rated with a low risk of bias (20, 37), two with moderate risk (17, 46), and three at severe risk of bias (23, 40, 43). Among the 18 retrospective studies, five were graded as having a serious risk of bias (4, 24, 26–28), and 13 (14, 18, 19, 21, 25, 29, 36, 42, 45, 47, 48, 50, 52) as moderate risk of bias. Four cross-sectional studies (5, 38, 39, 41) were judge to have a serious risk of bias (Table 5). Common reasons for loosing points in the quality assessment were poor or no description of the sample's diagnosis at baseline, differences in age variability among study groups, lack of appliance descriptions (wearing time, aligner change, mean number of aligners), and lack of standardization in study outcomes.

**Table 5:** Risk of bias in non-randomized studies (ROBINS-I tool).

First author, year	Bias due to confounding	Bias in selection of participants into the study	Bias in classification of intervention	Bias due to deviation from intended intervention	Bias to missing data	Bias in measurement of outcomes	Bias in selection of the reported results	Overall risk of bias
Bahammam et al., 2023 (48)	Low risk	Moderate risk	Moderate risk	Low risk	Low risk	Low risk	Low risk	Moderate risk
Blackham et al., 2020 (29)	Low risk	Moderate risk	Moderate risk	Low risk	Moderate risk	Low risk	Low risk	Moderate risk
Borda et al., 2020 (4)	Moderate risk	Seious risk	Moderate risk	Low risk	Low risk	Moderate risk	Low risk	Serious risk
Caruso et al., 2021 (24)	Low risk	Serious risk	Moderate risk	Low risk	Low risk	Moderate risk	Low risk	Serious risk
Chou et al., 2023 (42)	Moderate risk	Moderate risk	Moderate risk	Low risk	Low risk	Low risk	Low risk	Moderate risk
Cremonini et al., 2022 (26)	Low risk	Serious risk	Moderate risk	Low risk	Low risk	Low risk	Low risk	Serious risk
Cretella Lombardo et al., 2022 (45)	Low risk	Low risk	Moderate risk	Low risk	Low risk	Low risk	Low risk	Moderate risk
Cretella Lombardo et al., 2023 (19)	Low risk	Low risk	Moderate risk	Low risk	Low risk	Moderate risk	Low risk	Moderate risk
Cretella Lombardo et al., 2023 (28)	Low risk	Serious risk	Moderate risk	Low risk	Low risk	Low risk	Low risk	Serious risk
Dianiskova et al., 2021 (14)	Low risk	Moderate risk	Moderate risk	Low risk	Moderate risk	Moderate risk	Low risk	Moderate risk
Dianiskova et al., 2023 (41)	Low risk	Low risk	Moderate risk	Low risk	Low risk	Serious risk	Low risk	Serious risk
Favero et al., 2023 (43)	Moderate risk	Low risk	Moderate risk	Low risk	Low risk	Serious risk	Low risk	Serious risk
Gonçalves et al., 2023 (47)	Low risk	Moderate risk	Moderate risk	Low risk	Low risk	Moderate risk	Low risk	Moderate risk
Kong et al., 2023 (27)	Low risk	Serious risk	Moderate risk	Low risk	Low risk	Low risk	Low risk	Serious risk
Levrini et al., 2021 (18)	Low risk	Moderate risk	Moderate risk	Low risk	Low risk	Moderate risk	Low risk	Moderate risk
Lione et al., 2021 (17)	Low risk	Low risk	Low risk	Low risk	Low risk	Moderate risk	Low risk	Moderate risk
Lione et al., 2022 (40)	Low risk	Low risk	Moderate risk	Low risk	NI	Serious risk	Low risk	Serious risk
Lione et al., 2022 (46)	Low risk	Moderate risk	Low risk	Moderate risk	NI	Moderate risk	Low risk	Moderate risk
Lu et al., 2023 (20)	Low risk	Low risk	Low risk	Low risk	NI	Low risk	Low risk	Low risk
Ravera et al., 2021 (23)	Low risk	Low risk	Serious risk	Low risk	Moderate risk	Moderate risk	Low risk	Serious risk
Sabouni et al., 2022 (49)	Low risk	Moderate risk	Moderate risk	Low risk	Low risk	Low risk	Low risk	Moderate risk
Sauer et al., 2022 (39)	Moderate risk	Low risk	Moderate risk	Low risk	Moderate risk	Serious risk	Low risk	Serious risk
Sharma et al., 2021 (38)	Serious risk	Serious risk	Serious risk	Low risk	Low risk	Serious risk	Low risk	Serious risk
Shen et al., 2021 (36)	Low risk	Moderate risk	Moderate risk	Low risk	Low risk	Moderate risk	Low risk	Moderate risk
Sifakakis et al., 2018 (37)	Low risk	Low risk	Low risk	Low risk	Low risk	Low risk	Low risk	Low risk
Sun et al., 2022 (25)	Low risk	Moderate risk	Moderate risk	Low risk	NI	Low risk	Low risk	Moderate risk
Wang et al., 2023 (21)	Low risk	Low risk	Moderate risk	Low risk	Low risk	Moderate risk	Low risk	Moderate risk
Wu et al., 2023 (50)	Low risk	Low risk	Moderate risk	Low risk	Low risk	Low risk	Low risk	Moderate risk
Zybutz et al., 2021 (5)	Serious risk	Serious risk	Serious risk	Low risk	Low risk	Serious risk	Low risk	Serious risk

### 3.4 Study findings

#### 3.4.1 Dentoskeletal effects

Of the 32 studies, 21 analyzed the dentoskeletal effects of CA (4, 14, 17–21, 23, 24, 26–29, 36, 44–48, 50, 52).

##### 3.4.1.1 Transversal changes

Seven studies (17–21, 45, 48) evaluated differences in transversal maxillary arch width after expansion treatment with CA. Studies without control group (17, 18) showed significant increase in all linear interdental distances measured on digital models after treatment treatment with CA. Among the studies that compared CA with RME (19–21, 45) three reported increased transversal expansion after RME treatment compared to the CA group (19–21), accompanied by significant buccal tipping of the upper first molars. However, no significant differences were found in the CA group for this parameter. One study (19) pointed out a greater increase in the inter-canine width in the CA group compared to the RME group. In a previous study, the authors also found significative differences in morphological changes of the upper arch in the aligners group compared to the RME group (45).

When CA treatment was compared with the Quad Helix appliance (48), a retrospective study on CBCT demonstrated a significative decrease in bone height and width in the group treated with the Quad Helix.

The predictability of the transversal expansion following CA treated has been assessed in two studies without a control group (46, 47). Both studies supported that approximately 60% of the predicted expansion movement was achieved (46).

##### 3.4.1.2 Sagittal changes

Ten studies assessed sagittal dentoskeletal changes in Class II patients (14, 23–29, 49, 50) following CA + MA treatment. Three retrospective studies (26, 27, 49) without a control group, reported significative improvements in mandibular position after treatment with CA + MA in growing patients. However, the study by Sabouni et al. (49) pointed out only small changes the ANB angle, with no relevant changes in the SNB angle after treatment, suggesting that there were only minimal skeletal effects favoring Class II correction. The prospective study by Ravera et al. (23) compared the CA + MA treatment with untreated controls and supported increased correction of the ANB angle in the aligner group, particularly when the treatment was performed during the pubertal stage. Five retrospective studies (24, 25, 28, 29, 50) compared the effects of CA + MA with traditional functional appliances such as the Twin Block (TB), among others (50); of these studies, three also presented an untreated control group (28, 29, 50). Wu et al. (50) and Sun et al. (25) found more advanced mandibular position (SNB angle) in the TB group compared to the CA group. Caruso et al. (24) described significant differences between groups in the ANB angle after treatment, with more significative changes for the TB group, while the SNB angle increased similarly in both groups. The authors hypotheses that the difference was due to the increased retroclination of the upper incisors in the TB group compared to the CA group, as well as the difference in the mean ANB at the baseline. In contrast with these results, Cretella Lombardo et al. (28), showed no between-groups differences in the changes of the ANB angle after treatment.

Two retrospective studies (14, 42) evaluated the effects of Class II correction with intermaxillary elastics in the fixed multibracket (FMB) group compared to the CA group. Chou et al. (42) found that CA were more efficient in terms of treatment duration; furthermore, superimpositions indicated greater lower incisor proclination in the FMB group compared with the CA group. Dianiskova and colleagues (14) did not observe any statistically significant improvement in the sagittal skeletal relationship in the two groups, while a better control of lower incisors proclination was found with CA.

The reported effects of CA + MA on the inclination of maxillary and mandibular incisors are controversial. When compared with the TB appliance, three studies (24, 29, 50) found significantly increased retroclination of the upper incisors in the TB group and better control of the lower incisors in the CA group. Conversely, Sun et al. reported a significant reduction in the inclination of the upper incisors in both groups, with a greater difference observed in the aligner group, while lower incisor inclination increased similarly (25). Consistent with these results, the prospective study by Ravera et al. reported a significant reduction in the proclination of the upper incisors with CA + MA compared to untreated controls, when treatment was performed during the prepubertal stage of growth (23). Kong et al. also found that CA + MA treatment led to an average decrease of 3.44° in the inclination of the upper incisors, while the inclination of lower incisors increased by a mean of 2.62° (27). In contrast, Lombardo et al. (28) suggested that both appliances are effective in controlling incisor inclination during mandibular advancement. Cremonini et al. (26) also reported the control of upper and lower incisor inclination during treatment with CA + MA, although in a study without a control group.

##### 3.4.1.3 Crowding, OVJ and OVB

The retrospective study by Shen et al. (36), compared rate of overbite correction and alignment between CA and FMB, concluding that the effective rate was higher in the CA group. Conversely, the RCT by Merino da Silva and colleagues (44) demonstrated similar efficacy and efficiency for maxillary incisors crowding correction in mixed dentition between CA and fixed 2 × 4 mechanics. Borda and co-workers (4) pointed out similar effectiveness of CA compared to fixed therapy in terms of dentoalveolar correction, except for tooth alignment, overjet and occlusal relationship, which were significantly improved in the CA group. Caruso et al. (24) found overbite correction after treatment with CA + MA, while no differences were observed in the TB group. In contrast with these results, two retrospective studies (25, 28) showed that both CA + MA and TB appliances were able to reduce the overjet and overbite, with no differences between the groups. Finally, Wu and co-workers (50) reported that Van Beek Activator accounted the highest proportion of skeletal effects in reducing overjet (74.73%), compared to CA, TB, a Herbst appliance, and untreated controls.

#### 3.4.2 Oral health and periodontal changes

Six studies (11, 35–37, 44, 54) evaluated periodontal changes after treatment with CA in growing patients. The most commonly measured variables were the plaque index (PI), the gingival index (GI), the probing depth (PD), and the periodontal bleeding index (PBI). Two RCTs compared the effects of CA with FMB treatment (35, 44): Chhibber et al. (35) demonstrated no difference in periodontal health between subjects treated with CA, self-ligated brackets, or elastomeric-ligated brackets after 18 months of treatment. In agreement with these results, the RCT by Merino da Silva et al. (35, 44) reported similar PI during treatment both with both fixed 2 × 4 appliances and CA. In contrast, another RCT (11) and one prospective study (37), reported reductions in periodontal indices and bacterial levels, respectively, in the aligner group compared to patients treated with fixed appliances. Similarly, the retrospective study by Shen et al. (36) found that periodontal indices increased after treatment in both CA and fixed therapy groups, but the values in the between-group comparison were significantly higher in the FMB group. The cross-sectional study by Sauer et al. (39) showed that home oral hygiene with CA was intensified, and no dental plaque accumulation was observed. In the prospective study by Favero and colleagues (43) two experimental aligners with different edge designs were used to evaluate periodontal changes after three months. The results demonstrated that inflammatory indices worsened in the group with juxtagingival rims compared to vestibular rims.

#### 3.4.3 Quality of life, satisfaction and other outcomes

Five studies (5, 36, 38, 39, 41) evaluated the quality of life and satisfaction of CA treatment in growing patients. The case-control study by Sharma et al. adopted the Child Oral Health Impact Profile Short Form- 19 (COHIP-SF 19) and supplementary questions, concluding that there were no significant differences in mean quality of life and satisfaction between the CA group and the FMB group (38), after a minimum of six months of treatment. Similarly, the cross-sectional study by Sauer et al. (39) found that periodontal indices increased after treatment in both CA and fixed therapy groups, but the values in the between-group comparison were significantly higher in the FMB group. The cross-sectional study by Sauer et al. (5). The results highlighted that, although there were some differences between the treatment groups, their experiences with their appliance were overall comparable, and most patients in both groups reported high levels of satisfaction with their treatment.

However, the retrospective study by Shen et al. (36) showed that the quality of life and satisfaction were significantly higher in CA group compared to the FMB group, with a total satisfaction rate of 98.25% and 69.64%, respectively. Dianiskova et al. (41) founded similar results when comparing CA with the elastodontic therapy. Furthermore, one study retrospectively evaluated the treatment efficiency through questionnaires about the number of appointments, number of emergency visits, and treatment duration; all of these outcomes resulted in favor of the CA group compared to the FMB (4).

## 4 Discussion

Clear aligners (CA) have recently taken center stage in terms of their applicability and ability to successfully correct diverse types of malocclusions in all age groups, including early orthodontic treatments. The introduction of improved staging patterns, new aligner materials, and the implementation of hybrid therapies with different auxiliaries has increased the application of CA (55, 56). The aim of the present systematic review was to analyze and summarize the current scientific literature concerning the effects of CA treatment in children and adolescent patients. The main reported outcomes collected from the included studies were dentoskeletal effects, periodontal effects, quality of life, and satisfaction after CA treatment.

### 4.1 Dentoskeletal effects

#### 4.1.1 Transversal changes

In recent literature, some articles address the use of CA for the treatment of early transverse discrepancy. Two studies (17, 18) with moderate risk of bias found significant changes in transverse maxillary arch width after treatment with CA. However, in both studies, patients were recruited if they presented minor transversal discrepancy at the baseline. In fact, in the study by Lione et al. (17), 11 patients exhibited a crossbite involving one or two teeth, while the other 11 patients had no crossbite, and none presented a bilateral crossbite. Also, the studies included small sample sizes without a control group and had a short observational period (8 months), not accounting for possible relapse. Four studies (19–21, 45) evaluated transversal effects after expansion treatment with bonded RME compared to CA. The prospective study by Lu et al. (20), graded at low risk of bias, showed that RME allows a significantly greater expansion then CA, while CA produce dentoalveolar effects by delivering a certain amount of force on the dental crown. These results were supported by Cretella Lomardo et al., who highlighted that RME widened the palate to a greater extent (19), while CA induce maxillary arch shape modifications during expansion, in contrast with RME (45). Similarly, Wang et al. (21) reported that inter-canine width increased significantly in CA group compared to untreated controls, but the expansion amount was smaller than that achieved with SME. Three of these studies found significant buccal tipping of the upper first molars in the RME group, while no significant differences were found in the CA group (19–21). This was related to the possibility of planning an overcorrection of buccal root torque of the upper molars with CA treatment to avoid the side effects of dental tipping during expansion. In contrast with these results, the study by Bruni et al. (22) concluded that the more significant increase in intermolar width at the gingival level was observed in the RME group compared to the CA group, suggesting the occurrence of buccal tipping in the molar area using CA. However, all of the measurements of these studies were based on soft tissue and dental landmarks. Based on these evaluations, we can conclude that CA produce a certain amount of dentoalveolar expansion with the advantage of modifying arch form from early stages, and could be useful when mild transversal discrepancies are present. When skeletal expansion of the upper jaw is required, RME is considered more effective than CA, as it generates significantly higher forces leading to a predominantly skeletal effect (57). The greater magnitude of force produced by RME facilitates maxillary expansion by inducing structural changes in the bone, whereas CA primarily exert forces that are limited to dental movement. As a result, RME is particularly advantageous for addressing skeletal discrepancies and achieving substantial changes in maxillary morphology (57).

#### 4.1.2 Sagittal changes

Most of the included studies evaluated the sagittal skeletal effects of CA with MA in growing Class II patients with mandibular retrusion. Three retrospective studies without a control group observed some mandibular advancement in the short term (26, 27, 49). Differently, Sabouni et al. found that only the ANB angle significantly decrease (−0.55°) after treatment with CA + MA (49); however, the change was less than previously reported in the literature (23, 24). When comparing CA + MA with an untreated control group, Ravera et al. (23) found no differences in the SNB angle after treatment with CA + MA in the treated groups at different stages of growth, with significative changes only for the intermaxillary sagittal relationship in the CVM2 group (ANB −1.30°, *P* = 0.01) in the short time (18 months). A significant increase was noted in the CVM3 group regarding the linear growth of the mandible (Co-Gn +8.75 mm, *P* = 0.03). However, it is not clear how it is possible that no physiological mandibular growth occurred in the untreated control group (T0 = 113.24 ± 6.18; T1 = 113.07 ± 6.04). Five studies (24, 25, 28, 29, 50), graded at moderate or severe risk of bias, compared the effects of traditionally used functional appliances as the TB with those produced by CA + MA appliance. Caruso et al. (24) showed significantly higher decrease in the ANB angle in the TB group compared with the MA group, while the SNB angle increased significantly without differences between groups. However, the mean ANB value was significantly different between groups at the baseline. Furthermore, the TB group presented significant reduction in the SNA angle, which was related to the retroclination of the upper incisors, a finding not found in the CA group. In contrast, Sun et al. (25) demonstrated that SNB angle increase significantly only in the TB group, while the ANB angle and mandibular length (Co-Gn) were significantly different in both groups. However, the lack of comparison with an untreated control group, differences in age at the baseline between groups, and the short observational period represent limitations for considering these results reliable and to exclude the influence of natural mandibular growth. Conversely, three retrospective studies (28, 29, 50), graded at moderate and severe risk of bias, demonstrated significant changes in the ANB and SNB angles after both treatments with TB and CA + MA, with no differences between groups. In conclusion, the results obtained from this systematic review about sagittal effects of CA in correcting dentoskeletal class II are controversial. Thus, well conducted studies with large sample sizes and long-term follow-up periods are needed to establish the effectiveness of CA with MA compared to the traditional functional appliances.

#### 4.1.3 Dental effects

Interestingly, some included studies reported that CA provide good control of incisors inclination during sagittal correction of Class II malocclusion, both with MA (24, 26, 29, 50) and with intermaxillary elastics (14). In particular, the proclination of lower incisors is often an unwanted side effect of sagittal Class II correction, which is especially important in patients who already present increased proclination of lower incisors at the baseline before starting orthodontic treatment. Similarly, the retroclination of the upper incisors is frequently observed after Class II treatment, both with orthopedic devices and with fixed orthodontic appliances, and is often associated with retropositioning of the A point. The greater control provided by CA is likely associated with the intrinsic geometry of the aligner, which provides full coverage of the dental crown and maintains the entire dental arch through a unified structure (14, 51). Another possible explanation for the better control of lower incisor proclination might be linked to space management through digital setup: for instance, arch expansion, IPR, or the presence of preexisting spaces are conditions that offer the possibility for retroclination of the lower incisors. One more explanation could also be the incomplete correction of the curve of Spee. Authors have reported that controlling lower incisor inclination during Class II treatment offers promising effects in sagittal skeletal correction with CA and MA, since limited proclination of the lower incisors reduces the dentoalveolar compensation, thus providing more OVJ for guiding the mandible forward (49, 58).

### 4.2 Periodontal effects

The effects of CA on periodontal health have been evaluated in six studies, three of which were RCTs considered at low risk of bias (11, 35, 44). The 3-arm parallel-group prospective RCT by Chhibber et al. (35) found no evidence of differences in oral hygiene levels among CA, self-ligated brackets, and conventional elastomeric ligated brackets after 18 months of active orthodontic treatment. However, the short-term outcomes (after 9 months of treatment) show that the CA group participants had better GI and PBI scores than the fixed therapy groups (35). Similar findings were observed among adults when comparing CA with conventional multibracket therapy (59). Authors have reported that, when followed by a dental hygienist, patients undergoing orthodontic treatment with fixed appliances and CA do not show differences in gingival health. This was confirmed by the RCT of da Silva et al. (44), sustaining that there were no differences in plaque index in both treated groups in the short time (8 months). However, the value was very close to a level of significance in favor of a better oral hygiene for the CA group (44). Conversely, the preliminary RCT conducted by Abbate et al. (11) showed that during 12 months of orthodontic therapy, teenagers treated with removable appliances demonstrated better compliance with oral hygiene and presented less plaque and gingival inflammatory reactions as compared to their peers with fixed appliances. Similar results were reported in the retrospective study by Shen et al. (36) in a children population of 113 subjects, suggesting that bracketless invisible orthodontic treatment helps to improve periodontal health more than traditional fixed orthodontic treatment.

A recent systematic review authored by Di Spirito et al. (60) evaluated the long-term effects of CA compared to fixed multibracket therapy on periodontal health status, without age restrictions. The authors pointed out that CA provided slightly better control of PI and GI compared to fixed orthodontic appliances, especially in the short and medium terms, but no differences were found during the long-term follow-up (from the baseline to 12 months or more). Authors concluded that the impact of orthodontic treatment with CA and FMB on periodontal health should be considered comparable.

The meta-analysis conducted by Jiang et al. (53) in 2018 demonstrated that CA allowed relatively better periodontal health conditions (PI, GI, and PD) compared to fixed appliances, but the quality of evidence was medium. These findings are also in accordance with a previous review by Rossini et al. (61).

Therefore, it seems that while for adults no major differences are reported in terms of periodontal health, children and adolescents undergoing CA therapy exhibit better compliance with oral hygiene, reduced gingival indices, and improved periodontal status, especially in the short term.

### 4.3 Quality of life and satisfaction

Patient-Reported Outcomes Measures (PROMs) are the instruments used to assess information directly reported by the patient, without the interpretation of a clinician regarding their health, Quality of Life (QoL), or functional status associated with healthcare or treatment, among which satisfaction is one of the most important factors. Patient satisfaction and quality of life were examined in five studies (5, 36, 38, 39, 41). Sharma et al. (38) concluded that both the CA and fixed therapy groups were generally very satisfied with their treatment modalities. The overall quality of life of adolescent orthodontic patients undergoing treatment with fixed appliances and CA for a minimum of 6 months was comparable. Similarly, Sauer and colleagues (39) reported that oral health-related quality of life is only slightly affected during the first year of CA treatment in adolescents. These results are in line with previous findings by Flores-Mir et al. (62), who found that both the bracket-based and CA treated patients had statistically similar satisfaction outcomes across all dimensions analyzed in adults, except for the eating and chewing domain, in which the CA group reported more satisfaction. The retrospective study by Shen et al. (36) involving 113 children divided into two groups, concluded that CA treatment in children improves chewing function, quality of life, and satisfaction when compared with the FMB appliance. Similarly, a previous cross-sectional study by Azaripur et al. (63) demonstrated that patients treated with CA had greater satisfaction and reported less impairment in general well-being (6% vs. 36%) during orthodontic treatment than patients treated with fixed appliances. Zybutz et al. (5) compared CA with MA and TB appliance and reported that patients shared similar experiences for most of the parameters measured, but there were significant differences between the groups regarding appliance wear and management, discomfort, and function. A more recent systematic review (2023) by Kaklamanos et al. (6) assessing the patients’ health related quality of life following CA therapy, concluded that treatment with CA could be associated with better oral health related quality of life ratings compared to treatment with conventional labially placed metal fixed appliances. However, further high-quality studies are needed to reach safer conclusions.

## 5 Limitations

This systematic review highlights several limitations. The included studies are highly heterogeneous in design, patient characteristics, treatment protocols and outcomes, making comparisons difficult. Small sample sizes reduce statistical power and generalizability, while varying follow-up durations limit long-term data on treatment stability and effectiveness. Additionally, the lack of high-quality randomized controlled trials prevents drawing definitive conclusions about the relative effectiveness of early treatment with CA.

## 6 Conclusions

Based on the studies available in the literature, albeit the existing limitations, the following main conclusions about the effects of CA treatment in growing patients can be made:

• In the case of a mild transverse maxillary deficiency, CA produce dentoalveolar expansion during mixed dentition, but there is no evidence of skeletal effects.
• The effectiveness of CA + MA compared to traditional functional appliances in the correction of dentoskeletal Class II cannot be supported.
• There is no agreement in literature about the effects of CA on the upper and lower incisors; however, some studies reported that CA provide good control of incisor inclination during sagittal correction, when needed.
• In the short term, few studies support the notion that periodontal health and bacterial levels are better controlled in children and adolescents undergoing CA therapy compared to conventional fixed multibracket therapy.
• There are inconsistent findings to support that quality of life and patient satisfaction in growing patients are enhanced with invisible aligner therapy compared to fixed appliances.

These results suggest that early treatment with CA may be effective in certain type of malocclusions, but the evidence is inconsistent and does not always support advantages over traditional treatments, particularly regarding skeletal effects, Class II correction, and overall patient satisfaction.

## Data Availability

The original contributions presented in the study are included in the article/Supplementary Material, further inquiries can be directed to the corresponding author.
